# Haplotype analysis of *ApoAI* gene and sepsis-associated acute lung injury

**DOI:** 10.1186/1476-511X-13-79

**Published:** 2014-05-13

**Authors:** Jian Hao, Xian-Di He

**Affiliations:** 1 ICU Departments, First Affiliated Hospital of Bengbu Medical College, Bengbu 233004, China

**Keywords:** *ApoA1*, Polymorphism, Haplotype, Acute lung injury

## Abstract

**Background:**

Apolipoprotein A1 (*ApoA1*) is the major apoprotein constituent of high density lipoprotein (HDL) which exerts innate protective effects in systemic inflammation. However, its role in the acute lung injury (ALI) has not been well studied. In the present study we investigated the association between polymorphisms of *ApoA1* gene and ALI in a Chinese population.

**Methods:**

Three polymorphisms of the *ApoA1* gene (rs11216153, rs2070665, and rs632153) were genotyped by TaqMan method in 290 patients with sepsis-associated ALI, 285 patients sepsis alone and 330 age- and sex-matched healthy controls.

**Results:**

We found rs11216153 polymorphism of *ApoA1* was associated with ALI, the GG genotype and G allele was common in the ALI patients (76.9%, 88.1%, respectively) than both in the control subjects (55.8%, 75.8%, respectively) and in the sepsis alone patients (58.2%, 78.4%, respectively). Haplotype consisting of these three SNPs strengthened the association with ALI susceptibility. The frequency of haplotype GTG in the ALI samples was significantly higher than that in the healthy control group (OR = 2.261, 95% CI: 1.735 ~ 2.946, P <0.001) and the sepsis alone group (OR = 1.789, 95% CI: 1.373 ~ 2.331.P < 0.001). Carriers of the haplotype TTG had a lower risk for ALI compared with healthy control group (OR = 0.422, 95% CI: 0.310 ~ 0.574, P < 0.001) and sepsis alone group (OR = 0.491, 95% CI: 0.356 ~ 0.676, P <0.001).

**Conclusions:**

These results indicated that genetic variants in the *ApoA1* gene might be associated with susceptibility to sepsis-associated ALI in Han Chinese population.

## Introduction

Acute lung injury (ALI) and its more severe form, the acute respiratory distress syndrome (ARDS), are syndromes of acute respiratory failure that are characterized by acute pulmonary edema and lung inflammation. ALI remains an important cause of death in the intensive care units (ICU) and few specific therapies are available [1]. Although sepsis, pneumonia, aspiration, trauma, pancreatitis and multiple transfusion are recognized as the most common causes of ALI, only a small fraction of patients with these risk factors develop ALI [2]. Clinical and epidemiological studies have supported the hypothesis that genetic factors might play a part in the development and outcome of ALI [3-10]. Identification of genetic variants may provide new insight into the molecular pathogenesis of ALI and lead to the development of new diagnostic and therapeutic targets [6]. The pathogenetic basis of ALI is incompletely understood.

However, emerging evidence has suggested that the severity and outcome of ALI depend significantly on systemic inflammatory response [11]. Apolipoprotein A1 (*ApoA1*) is the major apoprotein constituent of high density lipoprotein (HDL) which exerts innate protective effects in systemic inflammation [12]. However, its role in the acute lung injury (ALI) has not been well studied. Previous study [13] suggested that one variant residing in the *ApoA1* gene, which involves a guanine to adenine transition 75 base pairs (bp) upstream from the start of transcription (G–75A) and destroys a site for the MspI restriction enzyme, was associated with ALI after cardiopulmonary bypass surgery. In their study, the authors only selected one single nucleotide polymorphism (−75 G > A) to perform a case–control study. And this is a hospital based case–control study, the selection bias cannot be avoidable and the subjects may not be representative of the general population.

In the present study, we established haplotypes of *ApoA1* gene consisting of 3 SNPs (rs11216153, rs2070665, and rs632153) and to assess the relationship between these haplotypes and ALI in a Chinese population.

## Material and methods

### Patients

The present study was reviewed and approved by the Ethics Study Board of First Affiliated Hospital of Bengbu Medical College. Informed written consent was obtained from all subjects or from their legal surrogates before enrollment. Definitions of sepsis and ALI were in accordance with the American College of Chest Physicians/Society of Critical Care Medicine Consensus Conference [14] and the American-European Consensus Conference statements (AECC) [15].

All sepsis subjects enrolled had either severe sepsis or septic shock. All patients were selected from the Emergency and Respiratory ICUs at First Affiliated Hospital of Bengbu Medical College, and were treated according to the Surviving Sepsis Campaign guidelines [16]. Exclusion criteria included age below 18 years, severe chronic respiratory disease, severe chronic liver disease (defined as a Child-Pugh score of > 10), using of high-dose immunosuppressive therapy and AIDS patients. All sepsis patients were screened daily for ALI/ARDS development and those who fulfilled the AECC criteria for ALI/ARDS were considered as ALI cases, which included ALI and ARDS patients; whereas those patients who did not develop ALI/ARDS during hospital stay were considered as sepsis alone patients. Baseline characteristics of all patients were obtained during ICU stay. Sex- and age-matched controls were selected from healthy blood donors. We selected the control subjects aged 55–66 years, which was matched to the patients group. There are not significant difference between the control subjects and the ALI patients in age and in sex.

Questionnaires including smoking, chronic illness and the history of ALI or sepsis were obtained from all control subjects. Healthy controls were defined as individuals without any recent acute illness, any chronic illness and a history of ALI or sepsis. To reduce the potential confounding from ethnic backgrounds, we only enrolled people with self-reported origin of central Han Chinese.

### Genotyping

**Figure 1:**
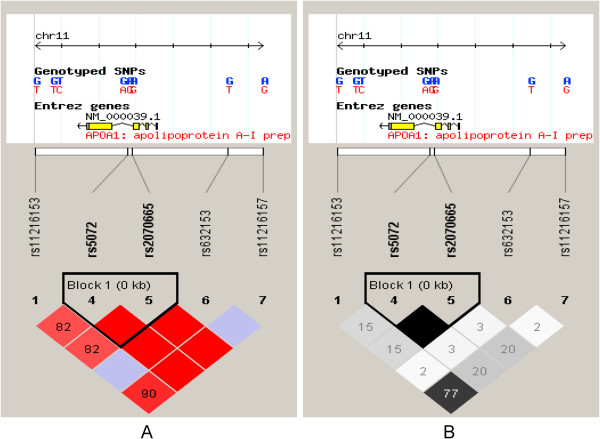
**Genetic variation at human *ApoA1 *gene.** Using the Haploview 4.2 software and the HapMap phrase II database, we scanned 5 genotyped single-nucleotide polymorphisms (SNPs) in Chinese Han. Linkage disequilibrium (LD) blocks across the locus in Chinese Han. LD block derived by solid spline method in Haploview 4.2. LD value shown: **(A)** |D’| × 100; |D’| colour scheme: |D’| =0: white; 0 < |D’| < 1: shades of pink; |D’| =1: red; **(B)** r ^2^ × 100; r ^2^ colour scheme: r ^2^ = 0: white; 0 < r 2 < 1: shades of grey; r ^2^ = 1: black.

There are 156 SNPs for the human *ApoA1* gene listed in the National Center for Biotechnology Information SNP database (http://www.ncbi.nlm.nih.gov/SNP). As shown in Figure 1, using the Haploview 4.2 software and the HapMap phrase II database, we obtained three tagging SNPs (rs11216153, rs2070665, and rs632153) for Chinese Han using minor allele frequency (MAF) ≥0.05 and linkage disequilibrium patterns with r ^2^ ≥ 0.6 as a cutoff.

Genomic DNA was extracted from the peripheral blood leukocytes using a DNA extraction Kit (Beijing Bioteke Co. Ltd). Genotyping was confirmed by TaqMan method as described previously [17].

### Statistical analysis

The SPSS 17.0 for windows was used for the statistical analysis. For each polymorphism, Hardy-Weinberg equilibrium was assessed using the standard χ^2^ test or Fisher’s exact test. Genotype frequencies in cases and controls were compared by χ^2^ tests. The genotype-specific risks were estimated as odds ratios (ORs) and it’s 95% CI. Based on the genotype data of the genetic variations, we performed linkage disequilibrium (LD) analysis and haplotype-based case–control analysis, using the SHEsis software (http://analysis2.bio-x.cn/myAnalysis.php) [18,19]. In the haplotype-based case–control analysis, haplotypes with a frequency of <0.03 were excluded. Statistical significance was established at P <0.05.

## Results

### Characteristics of the study population

The baseline characteristics of the study population are shown in Table 1. The primary source of infection was the lungs, involving 91.1% of the combined sample of sepsis alone and ALI patients. There was no significant difference in age, gender, BMI, diabetes, liver cirrhosis and history of smoking between ALI patients and sepsis alone patients (P >0.05).

**Table 1:** Demographic and clinical characteristics of subjects

	**Healthy controls**	**ALI patients**	**Sepsis alone patients**	**P value**
Number	330	290	285	N.A
Age, year	65.3 ± 10.2	63.8 ± 11.9	64.1 ± 10.7	0.643
Male (%)	186 (56.4)	172 (59.3)	170 (59.6)	0.877
BMI (kg.m^−2^)	21.1 ± 4.4	23.3 ± 4.8	23.1 ± 5.8	0.452
Diabetes (%)	0	56 (19.3)	51 (17.9)	0.078
Liver cirrhosis (%)	0	16 (5.5)	13 (4.6)	0.176
Smoker (%)	118 (34.2)	107 (36.9)	102 (35.8)	0.223
Sepsis insult (%)				
Lung (%)	N.A	265 (91.4)	262 (91.9)	0.989
Abdomen (%)	N.A	7 (2.4)	8 (2.8)	0.997
UTI (%)	N.A	5 (1.7)	4 (1.4)	0.879
Bloodstream (%)	N.A	11 (3.8)	10 (3.5)	0.987
Other (%)	N.A	2 (0.7)	1 (0.4)	0.774

### Associations of the ApoA1 gene SNPs with ALI risk

Table 2 shows the distribution of the genotypes and alleles of these 3 SNPs. The genotype distribution of each SNP did not show significant difference from the Hardy-Weinberg equilibrium values (data not shown). We found rs11216153 polymorphism of *ApoA1* was associated with ALI, the GG genotype and G allele was common in the ALI patients (76.9%, 88.1%, respectively) than both in the control subjects (55.8%, 75.8%, respectively, P < 0.001) and in the sepsis alone patients (58.2%, 78.4%, respectively, P < 0.001). However, we did not find any association of rs2070665 and rs632153 with ALI.

**Table 2:** Genotype distribution of ApoAI tag SNPs between case and control subjects

**SNPs**	**Genotype and allele**	**Control (n = 330)**	**ALI patients (n = 290)**	**Sepsis alone patients (n = 285)**	***P***^**a**^	***P***^**b**^
rs11216153	GG	184 (0.558)	223 (0.769)	166 (0.582)	<0.001	<0.001
	GT	132 (0.400)	65 (0.224)	115 (0.404)		
	TT	14 (0.042)	2 (0.007)	4 (0.014)		
	G	0.758	0.881	0.784	<0.001	<0.001
	T	0.242	0.119	0.216		
rs2070665	CC	101 (0.306)	80 (0.276)	83 (0.291)	0.594	0.812
	CT	181 (0.548)	161 (0.555)	159 (0.558)		
	TT	48 (0.146)	49 (0.169)	43 (0.151)		
	C	0.580	0.553	0.570	0.340	0.567
	T	0.420	0.447	0.430		
rs632153	GG	281 (0.851)	246 (0.848)	241 (0.846)	0.879	0.986
	GT	47 (0.142)	43 (0.148)	43 (0.150)		
	TT	2 (0.007)	1 (0.004)	1 (0.004)		
	G	0.923	0.922	0.921	0.983	0.931
	T	0.077	0.078	0.079		

*P*^a^ : ALI vs. control; *P*^b^ : ALI vs. Sepsis alone patients.

### Associations of the *ApoA1* gene haplotypes with ALI risk

As shown in Table 3 and Table 4, the frequency of haplotype GTG in the ALI samples was significantly higher than that in the healthy control group (OR = 2.261, 95% CI: 1.735 ~ 2.946, P <0.001) and the sepsis alone group (OR = 1.789, 95% CI: 1.373 ~ 2.331.P < 0.001). Carriers of the haplotype TTG had a lower risk for ALI compared with healthy control group (OR = 0.422, 95% CI: 0.310 ~ 0.574, P < 0.001) and sepsis alone group (OR = 0.491, 95% CI: 0.356 ~ 0.676, P <0.001).

**Table 3:** Distribution of haplotypes (ALI patients vs. healthy control)

**Haplotype**	**ALI patients**	**Control**	***P***	**OR (95% CI)**
G C G	276.00 (0.476)	332.00 (0.503)	0.339	0.897 [0.717 ~ 1.121]
G C T	45.00 (0.078)	51.00 (0.077)	0.983	1.004 [0.662 ~ 1.525]
G T G	190.00 (0.328)	117.00 (0.177)	<0.001	2.261 [1.735 ~ 2.946]
T T G	69.00 (0.119)	160.00 (0.242)	<0.001	0.422 [0.310 ~ 0.574]

**Table 4:** Distribution of haplotypes (ALI patients vs. Sepsis alone patients)

**Haplotype**	**ALI patients**	**Sepsis alone patients**	***P***	**OR (95% CI)**
G C G	276.00 (0.476)	279.99 (0.491)	0.602	0.940 [0.746 ~ 1.185]
G C T	45.00 (0.078)	45.00 (0.079)	0.931	0.981 [0.638 ~ 1.509]
G T G	190.00 (0.328)	122.01 (0.214)	<0.001	1.789 [1.373 ~ 2.331]
T T G	69.00 (0.119)	122.99 (0.216)	<0.001	0.491 [0.356 ~ 0.676]

## Discussion

In the present study, we found *ApoA1* gene polymorphisms and haplotypes were significant associated with ALI risk in a Chinese population. Some studies have been performed to find an association of genetic polymorphisms and ALI [20]. A prospective case–control study found that -607C/C genotype in IL-18 gene played a pivotal role in the development of ALI in Chinese Han population [21]. Another case–control study found that the IL-6 -572 polymorphism was associated with ALI [22]. Several studies have suggested that pre-B-cell colonyenhancing factor (PBEF) gene polymorphisms were associated with susceptibility and prognosis of ALI [23,24]. The plasminogen activator inhibitor-1 (PAI-1) 4G allele was associated with worse outcome in ALI/ARDS [25]. A prospective cohort demonstrated that the AC genotype at position −1221 in the NQO1 gene caused decreased transcription and was associated with a lower incidence of ALI following major trauma [26]. In a nested case–control study, patients with the NRF2 -617 A allele had a significantly higher risk for developing ALI after major trauma [27]. A case–control study found that myosin light chain kinase (MYLK) genetic variants were associated with increased risk of sepsis-associated ALI [28].

The *ApoA1* gene has recently been linked to many other diseases. A comparative study found that carrying the *ApoA1* -75 A allele could confer a higher risk of hyperlipidemia in obese children [29]. A prospective case–control study found that the *ApoA1* -75 G/A polymorphism influenced cholesterol metabolism [30]. A pilot study in a north Indian population suggested that the *ApoA1* -75 G allele might be a susceptibility allele for myocardial infarction [31].

In our study, we genotyped 3 SNPs in *ApoA1* in Chinese participants and assessed the association between *ApoA1* and ALI using a haplotype-based case–control analysis. The rs11216153 significantly differed between ALI patients and control participants, indicating that the risk of ALI is increased in participants with the G allele of rs11216153. Morris and Kaplan found that for genes with multiple susceptibilities, analysis based on haplotypes has advantages over analysis based on individual SNPs [32]. Consequently, in the present study, we successfully established haplotypes for the *ApoA1* gene from the different combination of the 3 SNPs. The frequency of the GTG was associated with increased risk for ALI. However, the TTG was associated with decreased risk for ALI.

Although we found rs11216153 was associated with ALI risk in the present study, this SNP was located in the non-coding region of *ApoA1*. Given that this SNP was tag SNP, it is more likely that rs11216153 is tagging other common or rare variants of the *ApoA1* gene associated with ALI. Another possibility is that the association might be due to LD with variants from nearby genes. Exhaustive resequencing is required to find or rule out the possibility of an as-yet-unidentified causal SNP in LD with rs11216153. And further functional studies are needed to investigate whether the variants have an effect on *ApoA1* mRNA stability and translatability.

This study has a number of strengths. First, a sepsis without ALI group was used for comparison to exclude the possibility of a false association with sepsis. Second, to minimize racial admixture, we focused on central Han Chinese patients, which could be regarded as one single homogenous population [23,24]. Third, to reduce the heterogeneous etiologies for ALI, the present study only included patients whose primary etiology for ALI was sepsis.

Some limitations of this study should be noted. First of all, these results should be interpreted with caution because the population was only from China, which reduces the possibility of confounding from ethnicity, so it does not permit extrapolation of the results to other ethnic groups. Second, the sample size of this study is relatively small, which may not have enough statistical power to explore the real association. Third, this is a hospital based case–control study, so the selection bias cannot be avoidable and the subjects may not be representative of the general population.

## Conclusion

In conclusion, the present results indicate that ALI is associated with the *ApoA1* gene polymorphisms. The GTG haplotype appear to be a risk genetic marker and the TTG haplotypes might be protective factor of ALI in Chinese people.

## Competing interests

The authors declared no competing interests exist.

## Authors’ contributions

JH and XDH carried out the molecular genetic studies and drafted the manuscript. JH carried out the genotyping. XDH performed the statistical analysis. Both authors read and approved the final manuscript.
